# Effect of Solubilizers on the Androgenic Activity of *Basella Alba* L. (Basellaceae) in Adult Male Rats

**DOI:** 10.15171/apb.2017.013

**Published:** 2017-04-13

**Authors:** Edouard Akono Nantia, Faustin Pascal Tsagué Manfo, Nathalie Sara E Beboy, Paul Fewou Moundipa

**Affiliations:** ^1^Department of Biochemistry, Faculty of Science, University of Bamenda, Cameroon.; ^2^Department of Biochemistry and Molecular Biology, Faculty of Science, University of Buea, Cameroon.; ^3^Laboratory of Pharmacology and Toxicology, Department of Biochemistry, Faculty of Science, University of Yaoundé I, Cameroon.

**Keywords:** Basella alba, Solubilizer, Androgenic activity, Male rat

## Abstract

***Purpose:*** Solubilizers play an important role in dissolution of pharmacological ingredients and should properly dissolve the active principle(s) while preserving its activities. This study investigated the effect of starch, gelatin, methylcellulose and polyvinylpyrrolidone 10000 in the preservation of the androgenic activity of the methanol extract Basella alba (MEBa).

***Methods:*** Different groups of male albino rats were orally given the MEBa (1 mg/kg) dissolved into either 1% gelatin (1% gel), %1 methylcellulose (1% MC) and 1% polyvinylpyrrolidone 10000 (1% PVP 10000) or 2% starch solution (2% SS) for 30 days. Thereafter, animals were sacrificed and serum testosterone and creatinine levels as well as alanine aminotransferase (ALT) activity determined. Vital and reproductive organs were dissected out and weighed, while liver thiobarbituric acid reactive substances (TBARS) and glutathione levels were determined.

***Results:*** Different treatments did not affect the animal body and organ weights. The MEBa stimulatory effect on testosterone production was preserved with 2% SS and 1% PVP 10000 as vehicles. Increased liver glutathione and TBARS levels were also observed in the animals fed with the MEBa dissolved in 2% SS and 1% Gel, respectively, while other biochemical parameters remained unchanged.

***Conclusion:*** Starch and polyvinylpyrrolidone 10000 stand as good preservation agents for MEBa androgenic activity, with starch exhibiting additional antioxidant activity through increase of glutathione levels.

## Introduction

*Basella alba* is a green leafy vegetable used as medicinal plant in folk medicine for treatment of male reproductive dysfunctions in West Region of Cameroon.^1^ Previous scientific studies showed that the active principles of *B. alba* were concentrated in the methanol fraction.^2,3^ Indeed, the stimulatory effect of the methanol extract of *B. alba* (MEBa) on testosterone release has been demonstrated in Leydig cells and male rats.^2,4,5^ The MEBa increased fecundity of rats exposed to flutamide *in utero* and alleviated maneb-induced deleterious effects on male reproductive function.^6,7^ Its antioxidant activity has been demonstrated using testicular homogenates. *B. alba* has also been shown to exhibit anti-inflammatory, hypocholesterolemic and antiatherosclerotic potetials.^8-10^ This plant is consumed as vegetable in other parts of the world including India, and phytochemical studies have revealed that it contains substantial amounts of antioxidant compounds such as ascorbic acid, carotenoids and phenolics.^11-13^ Consumption of the plant as food suggests that it may be safe, and the reported androgenic and antioxidant effects favor its use for treatment of male reproductive disorders attributed to androgen deficiency. However, efficient exploitation of the plant as phytomedicine, especially the active fraction MEBa, requires galenic formulation, which will enable proper delivery of the extract while conserving the desired pharmacological activity.

Solubility represents a key and limiting factor in search for suitable formulation, and even for testing compounds in animal experiments. Solubilizers are used to modify the polarity of water to allow an increase in the solubility of a nonpolar drug.^14,15^ Solubilizers should dissolve the pharmacological active ingredients, without interfering with the activity or affecting the host organism.^16^ Different vehicles commonly used to solubilize substances with pharmacological activity and apolar and polar extracts obtained from medicinal plants include polymers of carbohydrates, vinylpyrrolidone, etc.^12,13^ This study was designed to investigate the capacity of four vehicles namely starch, gelatin, methylcellulose and polyvinylpyrrolidone, to preserve the androgenic activity of the MEBa in adult albino male rats.

## Materials and Methods

### Materials

Animals used in this experiment were 2.5 -months-old Wistar albino male rats weighing 150 -200g. The animals were housed in plastic cages under standard conditions with 12-h photoperiod in the animal house of the Department of Biochemistry, University of Yaoundé I, Cameroon, and were given food and water *ad libitum*. The protocol of animal use was in compliance with ethical guidelines of the Cameroon National Veterinary Laboratory.

Fresh leaves of *B. alba* (identified at the Cameroon National Herbarium as specimen N° 40720) were collected in August 2007 in Dschang (West Region of Cameroon), dried at room temperature and ground into powder. The MEBa was obtained through successive extraction in hexane, methylene chloride and methanol, as reported elsewhere.^3^

Corn starch, gelatin, methylcellulose and polyvinylpyrrolidone 10000 were purchased from Sigma Aldrich (France). Other reagents were all of high quality grade.

### Methods

#### Study design

Forty eight rats were randomly assigned into 8 groups of 6 animals each. Four groups of animals were orally treated on a daily basis with 1 mg/kg suspended in 2% starch solution (2% SS), 1% gelatin (1% Gel), 1% methylcellulose (1% MC) and 1% polyvinylpyrrolidone 10000 (1% PVP 10000), respectively. The dose of 1 mg/kg was previously established as suitable dose for stimulation of testosterone release in male albino rats.^3^ The other 4 groups were treated with vehicle solutions (2% SS, 1% Gel, 1% MC, and 1% PVP 10000, respectively) and served as controls. The animals were treated for 30 days, and body weight recorded every other day. At the end of treatment, animals were sacrificed by decapitation and blood collected. The vital and reproductive organs (liver, kidneys, testes, epididymis, seminal vesicles and prostate) were dissected out and weighed. Sera were prepared from blood samples and used for determination of testosterone and creatinine levels as well as alanine aminotransferase (ALT) activity. Moreover, post-mitochondrial supernatant was obtained from liver tissues and used to quantify glutathione and thiobarbituric acid reactive substances (TBARS) levels.

#### Biochemical analyses

Testosterone quantification was carried out using a RIA commercial kit (P.A.R.I.S, Compiegne, France) according to instructions from the manufacturer. The inter - and intra-assay coefficients of variation were 5% and 8%, respectively, and the sensitivity was 4 pg per tube.

Serum alanine aminotransferase (ALT) activity was evaluated according to the colorimetric method of Reitman and Frankel, whereby the pyruvate resulting from the enzyme activity reacts with 2,4-Dinitrophenyl hydrazine to produce a hydrazone derivative, which in an alkaline medium produces a brown coloured complex whose intensity is measured at 530 nm.^17^ Creatinine levels were determined using the method of Bartels et al., which is based on the kinetic determination of the complex formed by creatinine andpicric acid in an alkaline medium.^18^ For the determination of thiobarbituric acid reactive substances (TBARS) levels in the liver post-mitochondrial supernatant, thiobarbituric acid was reacted with the analyte (TBARS), resulting into a pink colour complex which was measured spectrophotometrically at 532 nm. Ellman’s method was used for determination of glutahtione levels. The latter method is based on oxidation of GSH by the sulfhydryl reagent 5,5'-dithio-bis(2-nitrobenzoic acid) to form the yellow derivative 5'-thio-2-nitrobenzoic acid, which was measured in a spectrophotometer at 412 nm. The glutathione and TBARS levels were expressed relatively to protein levels in the supernatant, which were determined as described earlier.^19,20^

#### Statistical analyses

Assessment of the normality of data was conducted with the Kolmogorov–Smirnov test, and differences between groups assessed by the Student–Newman–Keul’s test. All analyses were performed using Sigmastat 3.1software (Systat Software Inc., San Jose, CA, USA), and a P value < 0.05 considered significant.

## Results

### Animal body and organ weights

**Figure 1:**
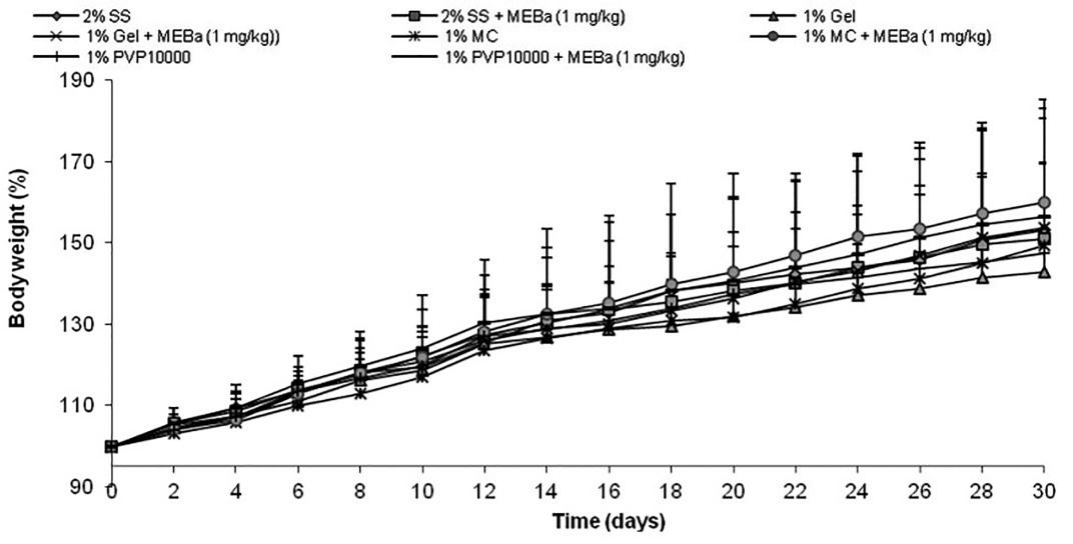
Relative body weight of animals treated with MEBa dissolved in different vehicles.
Each value represents the mean ± standard deviation from 6 animals per group. Animals were treated with MEBa (methanol extract of *B. alba*) dissolved in 2% starch solution (2% SS), 1% gelatin (1% Gel), 1% methylcellulose (1% MC) or 1% polyvinylpyrrolidone 10000 (1% PVP 10000), while control rats were given respective vehicles only.

**Figure 2:**
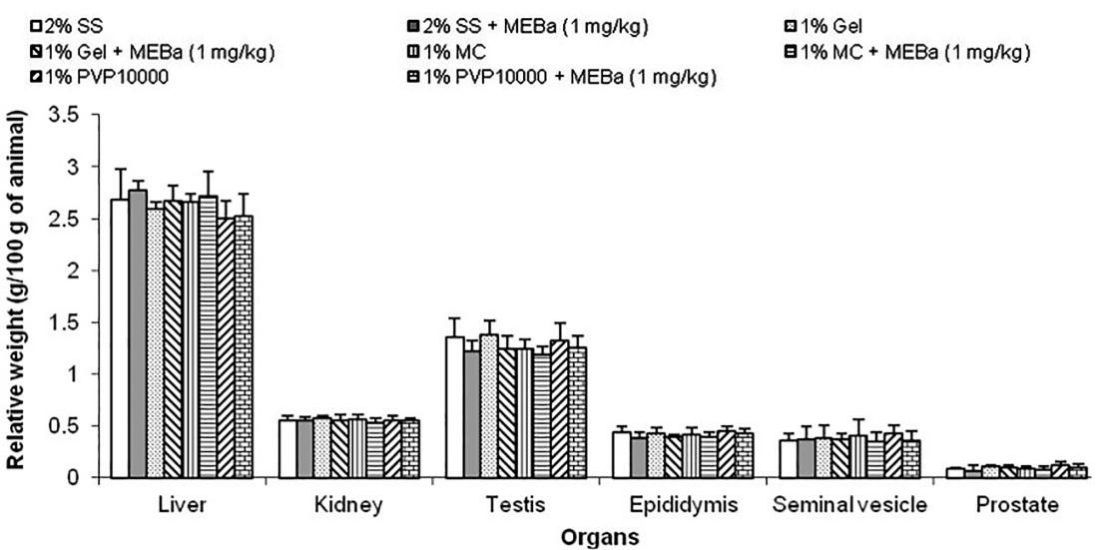
Relative organ weight of animal following treatment with MEBa dissolved in different vehicles. Each value represents the mean ± standard deviation from 6 animals per group. Animals were treated with MEBa (methanol extract of *B. alba*) dissolved in 2% starch solution (2% SS), 1% gelatin (1% Gel), 1% methylcellulose (1% MC) or 1% polyvinylpyrrolidone 10000 (1% PVP 10000), while control rats were given respective vehicles only.

The body weight of animals following 30 days treatment with MEBa dissolved in different vehicles is shown in Figure 1. All the animals gained weight over time, though there was no significant difference between control animals treated with solubilizers alone and rats receiving the MEBa dissolved in solubilizers. Likewise, weights of organs (liver, kidney, testis epididymis, seminal vesicle and prostate) from the animals remained unaffected by solubilizers (Figure 2).

### Serum testosterone level

**Figure 3:**
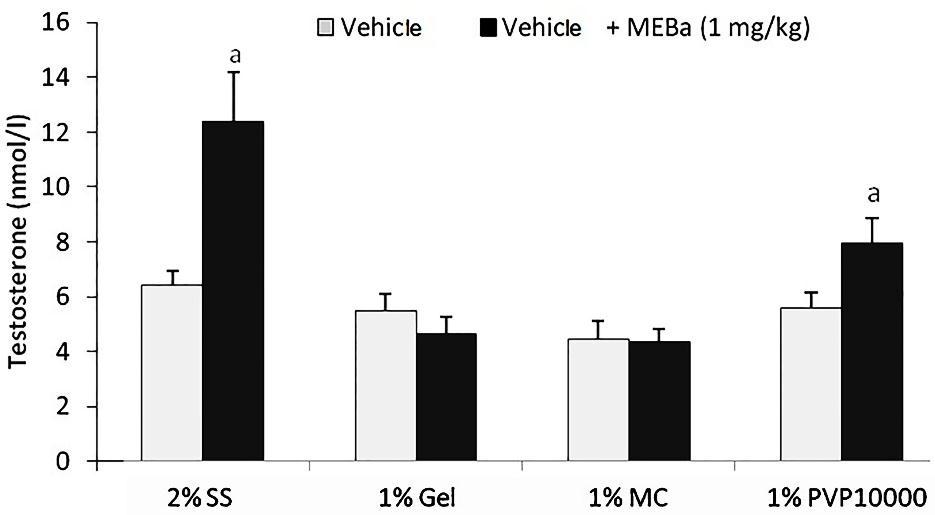
Testosterone levels treated with MEBa dissolved in different vehicles. Each value represents the mean ± standard deviation from 6 animals per group. Animals were treated with MEBa (methanol extract of *B. alba*) dissolved in 2% starch solution (2% SS), 1% gelatin (1% Gel), 1% methylcellulose (1% MC) or 1% polyvinylpyrrolidone 10000 (1% PVP 10000), while control rats were given respective vehicles only.^a^p<0.001 (Student-Newman-Keuls test).

As shown in Figure 3, the MEBa dissolved in 2% SS and 1% PVP 10000 significantly increased serum testosterone levels (P < 0.001) when compared to the respective controls or vehicle –treated animals. The increase in serum testosterone levels was more pronounced with the extract dissolved in 2% SS (155% higher when compared to testosterone levels in 1% PVP 10000 -treated rats).

### Biochemical parameters of liver and kidneys

Upon treatment of the animals, serum ALT activity and creatinine levels were determined, as well as liver TBARS and glutathione levels (Table 1). The obtained results showed that MEBa significantly increased glutathione (P < 0.02) and TBARS (P < 0.005) levels, when dissolved in 2% SS and 1% Gel, respectively. In contrast, serum ALT activity and creatinine levels remained unaffected.

**Table 1:** ALT activity and levels of creatinine, proteins, TBARS and glutathione in different animal groups

**Treatment**	**ALT (UI/L)**	**Creatinine (mL/L)**	**TBARS (nanomole/mg of proteins)**	**Glutathion (mmole/mg of proteins)**
2% SS	59.48±11.53	8.72±0.82	0.037±0.006	0.040±0.006
2% SS +MEBa (1 mg/kg)	61.35±8.55	8.34±1.06	0.045±0.015	0.058±0.003^a^
1% Gel	61.35±11.58	8.11±0.56	0.028±0.007	0.056±0.007
1%Gel + MEBa (1 mg/kg)	53.33±7.33	9.26±1.06	0.047±0.007^b^	0.067±0.012
1% MC	60.21±12.47	8.34±1.28	0.071±0.014	0.082±0.013
1% MC + MEBa (1 mg/kg)	49.37±6.35	9.11±0.90	0.075±0.014	0.086±0.007
1% PVP10000	52.29±10.94	8.95±0.48	0.061±0.013	0.080±0.006
1% PVP10000 + MEBa (1 mg/kg)	46.63±9.46	8.49±0.63	0.073±0.017	0.081±0.007

Each value represents the mean ± standard deviation from 6 animals per group. Assay animals were treated MEBa (methanol extract of *B. alba*) with 2% SS (2% starch solution), 1% Gel (1% gelatin), 1% MC (1% methylcellulose) or 1% PVP10000 (1% polyvinylpyrrolidone 10 000), while control rats were given respective vehicles of the MEBa.^a^p<0.02, ^b^p<0.005 (Student Newman Keuls test).

## Discussion

Solubility represents one of the key factors when investigating pharmacological effects of compounds or drug candidates in animal experiments. Solubilizing agents generally may possess effects that can interfere with the activity of the studied substance.^16^ Solubilizers are used to modify the polarity of water, in order to allow an increase in the solubility of a nonpolar compound.^14^ In this study, four polymers including two carbohydrate polymers (starch, methylcellulose), a protein-based polyemer (gelatin) and synthetic polymer (polyvinylpyrrolidone 10000) were used as vehicles to dissolve the MEBa and the androgenic activity of each preparation evaluated in adult male rats.

Following animals treatment, serum level of testosterone significantly increased in the animals given the MEBa dissolved either in 2% SS or 1% PVP 10000. PVP has been shown to increase the dissolution of some active ingredients and its use released the drug more quickly than gelatin or hydroxypropyl cellulose.^21,22^ However, 2% SS stand as the best solubilizer in terms of preserving the MEBa androgenic effect when compared to 1%PVP 10000. The androgenic effect of the MEBa dissolved in 2% SS was previously reported, and the current findings further emphasize on its ability to preserve activity of the bioactive ingredient(s) from the extract.^3,6,7^

Although other solubilizers including 1% Gel and 1% MC effectively dissolved the extract, these vehicles did not preserve the stimulatory activity of the MEBa on testosterone production. From these observations, it may be speculated that gelatin and methylcellulose hinder the activity of active ingredient(s) from MEBa through certain complex-formation that prevent absorption across the intestinal lining. These polymers may also inactivate the extract’s active ingredient(s) by triggering their degradation and result in the failure of the extract to stimulate testosterone synthesis. In fact, though methylcellulose and gelatin are commonly used in drug formulation, their negative effect on the kinetics of active principles has been suggested. It has been reported that methylcellulose is not absorbed by the intestines but passes through the digestive tract undisturbed when administered orally.^23^ Methylcellulose could also slow down the absorption extent of active substances due increased viscosity.^24,25^ Gelatin-based formulations usually encounter cross-linking which causes the formation of a swollen, very thin, tough, rubbery, water-insoluble membrane, also known as a pellicle. The pellicle acts as a barrier and restricts the release of the drug.^26^ Also MEBa contains various classes of phytochemicals including terpenoids, steroids, phenolics and saponins, and some of these phytochemicals (such as phenolic compounds) have been shown to form complex with gelatin through hydrogen bonds.^10,27^

The liver function in experimental animals was evaluated through determination of the activity of ALT in serum. ALT is a cytosolic enzyme from hepatocytes, and its activity increased in serum upon lysis or necrosis of the liver cells.^28^ ALT activity in the animals was not affected by the different solubilizers, when used either alone or in combination with the MEBa. The innocuity of MEBa when dissolved in 2% SS and used at the investigated dose (1 mg/kg) was previously reported.^3,5,6^ Interestingly, the latter formulation (MEBa dissolved in 2% SS) stimulated liver glutathione levels in the rats. Glutathione is well known for its importance in reducing reactive oxygen species molecules in order to neutralize their oxidative potential on living cells.^29^ Increase in glutathione levels in the animals’ liver suggests *in vivo* antioxidant activity of the MEBa, which is an added value to the androgenic effect. The activity of the MEBa in scavenging free radicals and its inhibiting effect on lipid peroxidation were previously demonstrated *in vitro*.^9^ Thanks to the increase in glutathione levels, *B. alba* may therefore have a protective capacity on animal cells/organs as reported earlier.^30^ Indeed, MEBa dissolved in 2% SS could protect male rats against pesticide (maneb) - induced reproductive toxicity.^7^ This antioxidant potential of MEBa when dissolved in 2% SS brings further justification of the beneficial effects of starch as solubilizer compared to polyvinylpyrrolidone and other polymers, including gelatin. Indeed, the use of gelatin as vehicle for MEBa administration also led to increased liver TBARS, which are products resulting from membrane lipids degradation.^31^ Gelatin consists of polyglycine which, because it has only the simplest amino acid side chain (-H) would not be expected to bring in any antioxidant activity.^32,33^ Therefore, the use of gelatin as vehicle for MEBa may thus have a detrimental effect on the integrity of membrane lipids in liver cells, though not important to cause significant cell lysis within the treatment period of 30 days, as ALT activity of animals remained unchanged. This is also supported by the organ weight and body weight which remain unchanged. The serum creatinine levels did not vary significantly between the assay and control animal groups, illustrating no negative effect of solubilizers and MEBa on renal function of rats.

Overall, the current study suggests that starch solution and polyvinylpyrrolidone preserved MEBa androgenic activity; with starch extract exhibiting an additional antioxidant effect. With preference given to starch, these two compounds can thus be considered as adjuvants in further steps towards development of a phytomedicine from MEBa that can be prescribed for treatment of male infertility related to androgen deficiency.

## Acknowledgments

This work was supported by the AUF doctoral fellowship program 2007 - 2009.

## Ethical Issues

The protocol of animal use was in compliance with ethical guidelines of the Cameroon National Veterinary Laboratory.

## Conflict of Interest

Authors declare no conflict of interest in this study.
